# Evolution of the Tri-PDZ Domain in PSD95 (DLG-4 Gene)

**DOI:** 10.1093/molbev/msaf309

**Published:** 2025-12-17

**Authors:** Riya Nilkant, Lisa Y Mesrop, Samuel Lobo, Onur Sakarya, Joan E Shea, Scott Shell, Soojin V Yi, Kenneth S Kosik

**Affiliations:** Department of Molecular, Cell and Developmental Biology, University of California Santa Barbara, Santa Barbara, CA 93106, USA; Department of Ecology, Evolution and Marine Biology, University of California Santa Barbara, Santa Barbara, CA 93106, USA; Department of Chemical Engineering, University of California Santa Barbara, Santa Barbara, CA 93106, USA; Department of Molecular, Cell and Developmental Biology, University of California Santa Barbara, Santa Barbara, CA 93106, USA; Neuroscience Research Institute, University of California Santa Barbara, Santa Barbara, CA 93106, USA; Department of Chemistry and Biochemistry, University of California, Santa Barbara, Santa Barbara, CA 93106, USA; Department of Physics, University of California, Santa Barbara, Santa Barbara, CA 93106, USA; Department of Chemical Engineering, University of California Santa Barbara, Santa Barbara, CA 93106, USA; Department of Molecular, Cell and Developmental Biology, University of California Santa Barbara, Santa Barbara, CA 93106, USA; Department of Ecology, Evolution and Marine Biology, University of California Santa Barbara, Santa Barbara, CA 93106, USA; Neuroscience Research Institute, University of California Santa Barbara, Santa Barbara, CA 93106, USA; Department of Molecular, Cell and Developmental Biology, University of California Santa Barbara, Santa Barbara, CA 93106, USA; Neuroscience Research Institute, University of California Santa Barbara, Santa Barbara, CA 93106, USA

**Keywords:** evolution, post-synaptic scaffold proteins, PDZ domains

## Abstract

Some genes encoding proteins within the co-evolved pre- and postsynaptic compartments are present in genomes long preceding the origination of the synapse within the animal kingdom. DLG4, gene encoding PSD-95, is one of the most abundant synaptic proteins. It is a MAGUK family member that shares a conserved domain structure comprised of one or multiple PDZ domains, a Src homology 3 (SH3), and a guanylate kinase (GK) domain. Here, we construct the phylogeny of the tri-PDZ domains in DLG4 to its deep ancestral origin in Filozoa, which includes animals and their nearest unicellular relatives. PDZ domain architecture appears to be a strong organizing feature of this gene lineage that originated with a single ancestral PDZ3-like domain in *Capsaspora owczarzaki* from which PDZ1 and PDZ2 were derived. The strong conservation of individual PDZ domain identities was captured by Evolutionary Scale Modeling (ESM2) across the boundary to the animal kingdom, corroborating distinct clades formed by the divergence of PDZ1, PDZ2, and PDZ3 in the phylogeny. CRIPT, PDZ3 ligand, is present in all Filozoa genomes studied here. AlphaFold2 Multimer demonstrates conserved binding function; however, conserved binding does not completely depend on either sequence motifs or hydrophobicity profiles. Rather, the most conserved feature is hydrogen bonds at the 0 and −2 positions of the ligand as an ancient foundational innovation for PDZ3 ligand interaction. Hydrogen bonds may loosen the sequence requirements for binding to allow a more extensive search space for protein-protein interactions that enhance fitness before the mutations that secure those interactions occur.

## Introduction

The multi-protein complexes of cellular machines are often assembled via binding through combinations of multi-domain proteins with diverse ligands. Assembling the component parts takes a very long time that in the case of the synapse preceded the origin of animals. The synapse is a particularly complex example of a cellular machine that requires the co-evolution of multiple protein interactions coordinately on both sides of the pre- and postsynaptic membranes. Here we focus on the evolutionary origin of the tri-PDZ domain among membrane-associated guanylate kinases (MAGUK) family members, some of which serve as key scaffolding proteins in post-synapse PDZ protein domains (postsynaptic density-95/discs large/zonula occludens) and are widely distributed across nearly all life forms including bacteria, archaea and fungi (Sakarya et al. 2010; Muley et al. 2019). Hundreds of distinct PDZ domains, each defined by their sequence, consist of 80 to 100 residues with a characteristic compact globular fold usually containing five to six antiparallel β strands and two α helices. Across phylogeny, particularly with the emergence of eukaryotes, PDZ domains frequently assume tandem contextually defined conserved architectures such that specific PDZ domains are positionally ordered in the protein and bind distinct ligands on a single scaffold. The modularity of PDZ domains makes them particularly amenable for the evolution of cellular machines in which multiple proteins have to coordinate their function and can link pre-existing components in novel ways. Among the PDZ domain-containing proteins are members of the MAGUK superfamily defined by their multi-domain structure consisting of PDZ (PSD-95/Dlg/ZO-1) domains, a Src homology 3 (SH3) and a catalytically inactive guanylate kinase-like (GK) domain. As a scaffold, they tether membrane receptors to intracellular signaling pathways. The number of PDZ domain copies varies among MAGUK family members. The most abundant PDZ domain protein in the synapse is PSD-95 (encoded by the DLG4 gene), which accounts for most of the mass in the postsynaptic density. The tri-PDZ scaffold plays a key role in assembling protein complexes and regulating synaptic transmission in neuronal excitatory synapses (Craven and Bredt 1998) by binding to neuronal receptors (NMDA), coordinating stimulus-induced recruitment of AMPA receptors to synapses, and co-localizing downstream effectors such as nNOS as well as promoting signals that fan out to multiple outputs from the assembled complex. In *Drosophila melanogaster*, DLG is involved in synaptic clustering of Shaker potassium channels (Tejedor et al. 1997), junction structure, cell polarity, and localization of membrane proteins.

PSD-95 contains three tandem PDZ domains (PDZ1, PDZ2, PDZ3), along with an SH3 domain and a GK domain. The PDZ domains of this tri-PDZ domain protein can each be uniquely identified by their position within the overall protein architecture. The context-dependent identities maintain an exquisite level of purifying selection such that each of the three PDZ domains in PSD-95 have conserved their positional identity from sponge to human (Sakarya et al. 2007). Ancestral PDZ domains in some protista MAGUK orthologs show architectural conservation of individual PDZ identities.

PDZ-binding proteins generally share a C-terminal motif that has been classified into three types with the consensus sequences: S/T-X-hydrophobic-COOH for type I, hydrophobic-X-hydrophobic-COOH for type II, and D/E-X-hydrophobic for type III (von Nandelstadh et al. 2009). Hydrophobic residues are V, I, L, F, W, Y, and M. The binding of *CRIPT* (CXXC repeat containing interactor of PDZ3 domain (Niethammer et al. 1998) to PDZ3 is well studied with a solved structure (Doyle et al. 1996). The affinity of CRIPT (K_D_ in the low µM range for the *Homo sapiens* CRIPT:DLG4 PDZ3 interaction) resides in the six last amino acid residues (Saro et al. 2007; Gianni et al. 2011; Toto et al. 2016) which corresponds to the consensus PDZ-binding sequence (Tonikian et al. 2008). The functional changes that accompanied and enabled the evolution of complex molecular machines remain unresolved. To gain insights into this problem here we integrated evolutionary and modeling approaches.

Here, we use phylogenetic analysis, ancestral sequence reconstruction, protein structure predictions, and protein language modeling with Evolutionary Scale Modeling (ESM) for ligand binding predictions to infer the molecular properties of the ancestral PDZ domain within the DLG4 clade and the evolutionary changes that occurred in the binding interactions of ancestral PDZ domains with the potential for new synaptic ligands. To infer the origin and evolution of the tri-PDZ domain architecture, we performed a phylogenetic analysis of the MAGUK gene family and expanded on previously reported MAGUK phylogeny by including proteomes from the unicellular choanoflagellate, *Salpingoeca rosetta*, and two Ctenophore species *Bolinopsis infundibulum* and *Mnemiopsis leidyi*. Thus, we trace the origin of the tri-PDZ domain to the protista, well before the origin of animals. By comparing PDZ domains in unicellular relatives and Metazoans, we found PDZ3 is ancestral to PDZ one and two and originated along the ancestral lineage leading to the Holozoan clade. The structure and physicochemical properties of these proteins were evaluated by conducting molecular dynamics simulations solvating the PDZ protein and analyzing the hydrophobicity pattern based on tetrahedral waters (Robinson Brown et al. 2023; Jiao et al. 2024).

## Results

### The MAGUK Gene Tree Captures the Origin and Evolutionary History of the Ancestral PDZ Domain in the DLG-4 Subfamily

**Fig. 1.**
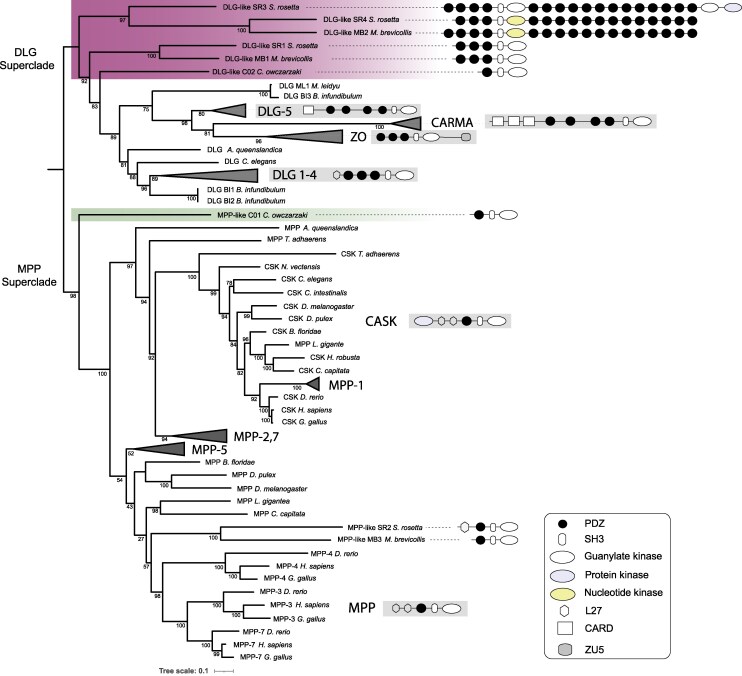
Phylogeny of SH3+GUK domains of DLG 1-4 subfamily and other MAGUK genes. Phylogenetic reconstruction was performed using IQTree. Bootstrap support values (*n* = 1,000) are given for all nodes. Superclade and collapsed clade names are indicated in bold. The domain architecture is shown for MAGUK gene families, including DLG-like homologs and MPP-like homologs in single-celled relatives *C. owczarzaki*, *M. brevicollis,* and *S. rosetta*. PDZ domains for DLG1-4 architecture are numbered and colored according to the structure-based nomenclature, with PDZ1 as the first N-terminal PDZ domain.

To understand the evolutionary relationship between individual PDZ domains we generated a comprehensive PDZ domain phylogeny by extracting all PDZ domains of the DLG-4 gene and its homologs from three unicellular Holozoans and several representative Metazoan species. The phylogenetic analysis of SH3+GUK domains from across major classes of the MAGUK family included DLG1-4, MAGI, ZO, CARMA, DLG5, MPP1, MPP2-7, MPP5, and CASK. Our comprehensive SH3+GUK domain analysis corroborated the previously proposed two superclades (Fig. 1) de Mendoza et al. (2010). Following the nomenclature reported in de Mendoza et al. (2010), the first clade was labeled “DLG super class,” which consists of DLG5, DLG1-4, ZO, and CARMA subfamilies and the second clade was labeled the “MPP super class,” which comprises MPP1, MPP2-7, MPP5, and CASK. In addition to the *Capsaspora owczarzaki* and *Monosiga brevicollis* DLG-like homologs previously reported in Mendoza et al. (de Mendoza et al. 2010), we found additional DLG-like orthologs present in the choanoflagellate *S. rosetta* (Fig. 1, pink clade). We also identified two genes that are closely related to the MAGUK family, one in *S. rosetta* and the other in *M. brevicollis*, that possess a PDZ and SH3+NK domain architecture, instead of the typical MAGUK core architecture of SH3+GUK (Figure S3 and Table S4). The presence of the NK domain related to a wide variety of roles in nucleotide metabolism, the biosynthesis of coenzymes and aromatic compounds, as well as the metabolism of sugar and sulfate will likely alter the function of the scaffold (https://www.ncbi.nlm.nih.gov/Structure/cdd/cl17190). We also found a total of six DLG-like homologs across two Ctenophore genomes, *B. infundibulum* and *M. leidyi*. We did not find any DLG-like homologs with the MAGUK core architecture (PDZ, SH3, and GUK) in any of the available transcriptomes or genomes of Teretosporea, Fungi, Amoebozoa, and Bacteria suggesting this domain architecture was a Holozoan innovation. The MAGUK gene tree captures the origin of DLG-4 architecture, as the absence of DLG-like homologs in representatives predating the divergence of Filasterea and Choanoflagellates suggests the unique PDZ, SH3, and GUK architecture originated in the common ancestor of the Filasterea and Choanoflagellates. Our results are consistent with the idea that within the DLG superclass, the MAGUK core architecture was further adapted in Choanoflagellates and gave rise, after duplication of the most C-terminal PDZ domain, to DLG, ZO and CARMA subfamilies.

### A Tri-PDZ Domain Architecture Lies at the Origin of the DLG-4 Phylogeny

**Fig. 2.**
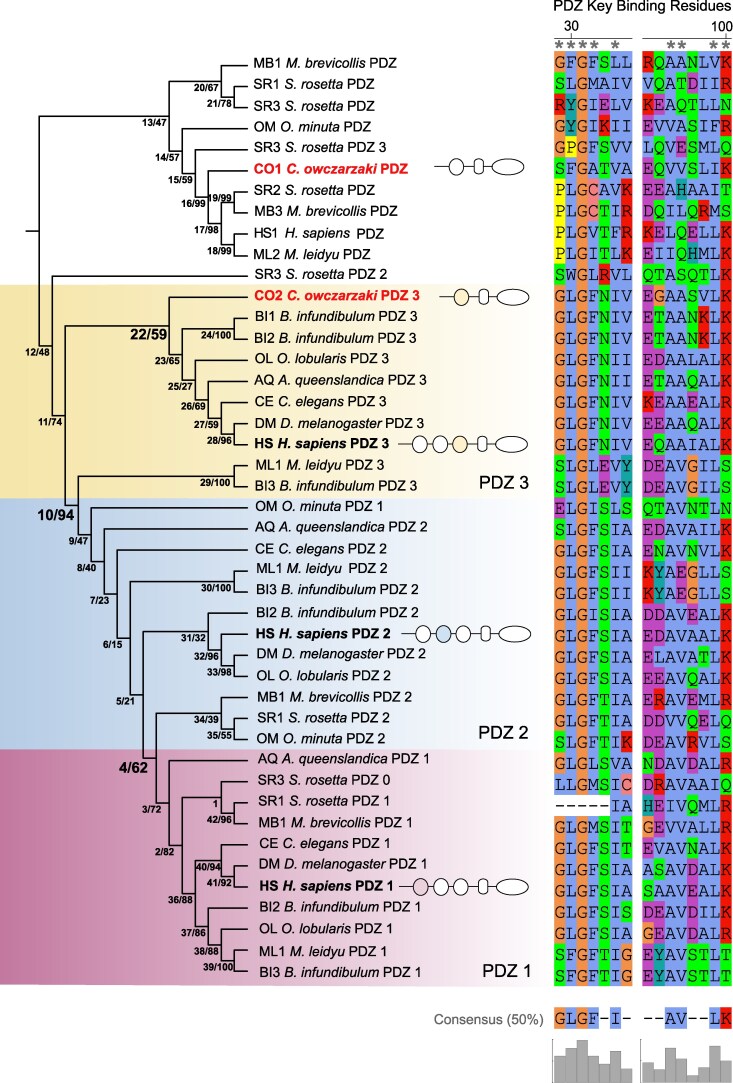
Phylogeny of PDZ domain sequences of *H. sapiens* DLG4 gene and DLG4-like homologs in single-celled organisms. The topology and branch lengths were obtained by maximum likelihood analysis using IQ-TREE2. The tree is midpoint rooted. The ancestral node numbers, indicated by the first number before the slash, are followed by the bootstrap scores (*n* = 1,000) for each node, represented by the number after the slash. The two *C. owczarzaki* DLG4-like homologs, CO1 and CO2, are labeled in bold red. The three PDZ domains in *H. sapiens* DLG-4 are in bold black and labeled according to the structure-based nomenclature, with PDZ1 as the first N-terminus PDZ domain, PDZ2 at the second position and PDZ3 at the third position before the start of the SH3+GUK domains. We labeled the clades following the same structure-based nomenclature for each PDZ in *H. sapiens* DLG-4: PDZ1 clade (light pink), PDZ2 (light blue), and PDZ3 (light yellow). Note that the CO1 clade typically has single PDZ domains except for SR3, which uniquely has four PDZ domains (see text). To avoid confusion, the PDZ domains in genes with single domains within the C01 clade are not labeled as “3” and are labeled with just “PDZ”. This distinguishes them from the ancestral PDZ domain labeled “3” which is orthologous to PDZ 3 in the C02 gene. To the right of the phylogeny, we display a subset of the PDZ domain alignment and mark PDZ key binding residues with asterisks.

The tri-PDZ architecture of DLG-4 traces its origin and subsequent diversification from an ancestral PDZ domain in the protista lineage, *C. owczarzaki*. To understand the evolutionary history of the tri-PDZ domains in the DLG1-4 subfamily, specifically PSD95 (DLG-4), we extracted all the N-terminally positioned PDZs of the DLG-4 gene and its homologs from three unicellular Holozoans, *M. brevicollis* (MB), *S. rosetta* (SR), *C. owczarzaki* (CO), and seven Metazoan species. Specifically, we extracted all the PDZ domains N-terminal to the SH3+GUK domain, and labeled them according to the structure-based nomenclature, with PDZ1 as the first N-terminal PDZ domain, PDZ2 as the second and PDZ3 as the third N-terminal PDZ domain, positioned at the start of the SH3+GUK domain (Fig. 2). From this set of 44 PDZ domains, we generated a phylogeny to determine the origin and diversification of the PDZs as they related to DLG1-4 subfamily architecture. Interestingly, we identified a DLG-like homolog in *S. rosetta* (SR), designated as SR3 (Fig. 1), which contains an additional PDZ domain at the N-terminal position, referred to as PDZ0 (Fig. 2) and falls within the PDZ1 clade.

The phylogeny indicates that the single PDZ domain found in CO2 is ancestral to other PDZ domains. In addition, the PDZ3 domains are ancestral to both PDZ1 and PDZ2, as PDZ3 is shared with both Filesterea and Choanoflagellates, while the other two PDZ domains appear after the divergence of Filesterea and Choanoflagellates (Figs. 1 and 2, and Figure S4). The single PDZ domain from the CO1 and CO2 group with the ancestral PDZ and the PDZ3 domains, respectively (shown in red in Fig. 2). In particular, the single PDZ domain of CO2 and *H. sapiens* DLG-4 PDZ3 form a monophyletic group with a bootstrap value of 60% which suggests that the PDZ domain in *C. owczarzaki* CO2 is orthologous to *H. sapiens* DLG-4 PDZ3 and ancestral to PDZ1 and PDZ2 clades. Due to the orthologous relationship between *C. owczarzaki* CO2 PDZ and *H. sapiens* DLG-4 PDZ3, and that *C. owczarzak*i CO2 PDZ is ancestral to PDZ1 and PDZ2 clades, we labeled the single PDZ domains in CO2 as PDZ3 (Fig. 2). The second *C. owczarzaki* gene, CO1, is the outgroup to MPP subclade (Figs. 1 and 2) and based on its distinct PDZ domain forms a separate clade. Together with VAM-1 (Fig. 2) in human, these single PDZ domains followed by an SH3 domain and a GUK domain (Tseng et al. 2001) form a distinct molecular crown group within the MAGUK family. PDZ domains within the CO1 clade (Fig. 2) exhibit distinct key binding residues compared to those of DLG genes, consistent with clade-specific divergence observed in other PDZ families (Sakarya et al. 2010).

The divergence of PDZs in positions 1, 2, and 3 into distinct clades (PDZ 1, 2, and 3) does not result in strictly monophyletic clades (Fig. 2), yet their approximate clustering by position highlights the importance of their architectural arrangement within the gene, suggesting that the observed pattern of strong conservation may have been shaped by selection pressures on the local position of specific binding interactions (Fig. 2). *S. rosetta* is an exception to this domain architecture. The SR genome has two DLG-like homologs designated SR1 and SR3, and one MPP-like homolog designated SR2, which contains a single PDZ domain as is typical for the MPP clade. SR1contains a single PDZ domain, not the expected tri-PDZ domain and this domain most closely resembles PDZ3. SR3 is a DLG-like homolog (Fig. 1) with three PDZs that, curiously, are more closely related to the single PDZ domain in C01 (MPP-like) than C02/DLG-4 PDZ 3 in Fig. 2. The PDZ domains in SR3 labeled PDZ 0 are similar to DLG-4 PDZ1. Unlike the other members of the gene family where the domain architecture seems to be conserved, SR3 took a different evolutionary route that appears related to domain duplication and shuffling.

### Ancestral Reconstruction Reveals Conservation of Key PDZ Binding Residues Across Distantly Related Species

**Fig. 3.**
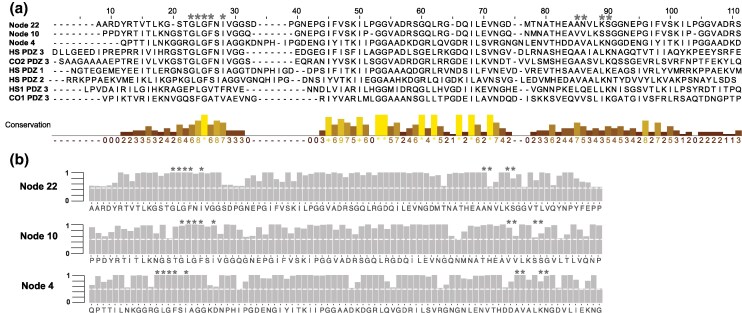
The three ancestral PDZ sequences were reconstructed with high confidence and with mean posterior probabilities >0.5. a) All PDZ sequences used in Fig. 2 were included in the ancestral sequence reconstruction. Multiple sequence alignment of ancestral nodes—Node 22, Node 10, and Node 4—reveals similarity in nine key binding residues with extant *H. sapiens* DLG-4 PDZ1, 2, and 3, as well as *C. owczarzaki* CO2 PDZ but shows significant differences from *C. owczarzaki* CO2 PDZ3 and *H. sapiens* VAM-1 PDZ3. Residues of the nine key binding residues are highlighted in blue. b) Posterior probabilities of the reconstructed ancestral PDZ sequences are shown for all three ancestral nodes. The dashed white line indicates the posterior probability of 0.5, which is the threshold for identifying ancestral sites. The PDZ key binding residues are denoted with asterisks.

To determine whether the key PDZ-binding residues were present before and after the diversification of Choanoflagellates and Metazoans, we used ancestral sequence reconstruction to infer the ancestral PDZ domains (see Methods). Based on the PDZ domain phylogeny, we identified the maximum posterior (Bayesian inference maximum) PDZ ancestral sequences for each node in the PDZ phylogeny (Fig. 2). We focused on three ancestral nodes that are key to understanding the origin of PDZ residues, which have been functionally demonstrated to be important for binding. We focused on the ancestral PDZ at the origin of Filasterea and Choanozoa (Choanoflagellates+Metazoans) clades (Node 22; Fig. 2), the ancestral PDZ before the evolution of PDZ1 and PDZ2 clades along the ancestral lineage leading to Choanoflagellata, over 700 million years ago (Node 10; Fig. 2) and the ancestral PDZ before the divergence of PDZ1 and PDZ2 clades (Node 4; Fig. 2). We then examined the nine key PDZ-binding residues (https://www.ncbi.nlm.nih.gov/Structure/cdd/wrpsb.cgi), consisting of GLGF (Songyang et al. 1997) in the positions 23 to 26 in position 28, and AA and LK in positions 85 to 86 in the human PDZ3 domain (Fig. 3a), The node 22 within the PDZ3 clade encompassing PDZ domains from diverse species shows the conservation of the first five of these nine residues, but not the last four residues. The node 10 in the common ancestor of PDZ2 and PDZ1 domains encode V at the conserved V position. Finally, the node 4, near the origin of PDZ1 domain, harbors the same amino acids in eight out of nine key residues. These observations indicate a gradual emergence of key residues during the evolution of distinctive PDZ domains (Fig. 3). In comparison, PDZ domains in distantly related HS1 (VAM-1) and CO1 are divergent from the functional modern PDZ domain residues (4/9 and 2/9 residues, respectively) (Figs. 2 and 3). Correspondingly, the nine key PDZ-binding residues between orthologous *C. owczarzaki* CO2 PDZ3 and *H. sapiens* DLG-4 PDZ3 domains remained similar (Fig. 3).

### ESM2 Language Model Reflect the Evolutionary Relationships Between the PDZ Domains

**Fig. 4.**
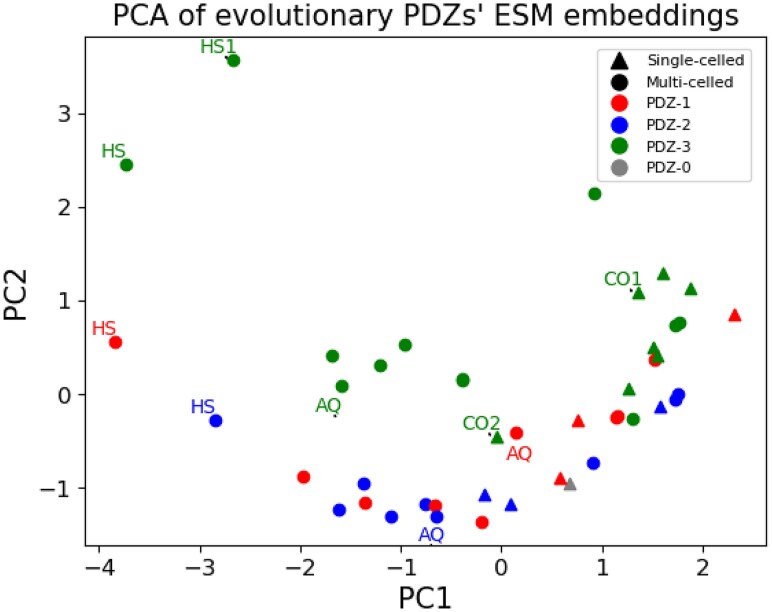
Plots of ESM2 15B for genes in the SH3+GUK phylogeny. PCA plot of ESM embeddings. Key: Gray: PDZ0, Red: PDZ1; Blue: PDZ2; Green: PDZ3; Triangle: single-celled; Circle: multi-celled. PC1 captures 25% variance and PC2 captures 11% variance. Key for naming convention: HS: *H. sapiens*, AQ: *A. queenslandica* CO: *C. owczarzaki*. The full annotated plot can be found in Figure S1.

Mutations that have been selected over evolutionary time scales reflect biological structure and function. These evolutionary patterns can be learned using transformer-based protein language models that predict masked-out amino acids in its training (Lin et al. 2023), similarly to the popular large language models that predict masked-out words (Brown et al. 2020). This approach does not rely on multiple sequence alignments, and therefore can independently investigate the phylogenetic relationships of the MAGUK PDZ domains described above. From the ESM2 protein language model, we generated vectors of embeddings for each domain (by averaging the per-residue embeddings (Rives et al. 2021)). These embeddings are rich protein representations that capture structural and functional relationships between amino acids, even enabling structure prediction (Lin et al. 2023). We performed a principal component analysis (PCA) on the ESM-2 embeddings (Fig. 4). 5,120 embeddings were calculated per residue using the 15B parameter of the ESM2 model. The ESM embeddings reflect some trends seen in the sequence-based phylogeny from Fig. 2. For example, single-celled organisms (Choanoflagellates and *C. owczarzaki*) were exclusively positioned to the right of the zero in PC1. PC2 separates some PDZs from other PDZ domains (PDZ 3 from 1 and 2). These observations indicate that the ESM2 embeddings harbor information regarding the distinctive PDZ domains, as well as reflecting some differences between single-celled vs. multi-cellular organisms.

To evaluate these observations, we performed comparisons of ESMs using the analysis of variance with Tukey post hoc test (Tukey HSD) for 10 PCs. PC2 showed significant differences between PDZ3 and PDZ1 as well as PDZ3 and PDZ2 (*P* < 0.01 for both comparisons, Post hoc Tukey HSD). Additionally, PC7 also captures distinct PDZ groupings between PDZ 1 vs 2 (*P* < 0.01, post hoc Tukey HSD), 1 vs 3 (*P* < 0.01, post hoc Tukey HSD), and 2 vs 3 (*P* < 0.05, post hoc Tukey HSD) (Table 1). We also tested differences between single-celled vs. multi-celled organisms using Welch's two tailed *t*-test across ten principal components. Embeddings for the single-celled organisms significantly differed from multi-celled organisms along PC 1, 4, and 6, with the single-celled organisms toward the right along PC1 and multi-celled organisms on the left along PC1 (*P* < 0.01). While general trends between PDZ divergence and evolution were corroborated in the separation of PDZs between single vs multi-cellular organisms, notable differences within clades were observed in the PCA plots. HS1 (Human VAM-1) and CO1 share the same architecture of a single PDZ followed by an SH3 and GUK domain and group in the same clade in Fig. 2. In the ESM PCA plot, HS1 appears closer to HS PDZ3 along both PC1 and PC2 while CO1 appears distinct, unlike the phylogeny. CO2 groups with HS PDZ3 in the phylogeny and shares conserved binding residues (GLGF); however, it varies along both PC1 and PC2. Ancestral nodes were not included in this analysis due to low bootstrap scores.

**Table 1.** *P*-values for differences among PDZ proteins in single vs multi-cellular organisms

ANOVA test for PDZ types		Post hoc Tukey HSD	
PC (% Variance)	Anova *P*-value	Tukey HSD *P*-value	
PC1 (24.5%)	*P* = 0.86	—	
**PC2 (11.0%)**	*P* < 0.01*	PDZ 1 vs 2	*P* = 0.46
		PDZ 1 vs 3	*P* < 0.01*
		PDZ 2 vs 3	*P* < 0.01*
PC3 (8.1%)	*P* = 0.07	—	
PC4 (5.0%)	*P* = 0.18	—	
PC5 (4.4%)	*P* = 0.43	—	
PC6 (3.8%)	*P* = 0.87	—	
**PC7 (3.5%)**	*P* < 0.01*	PDZ 1 vs 2	*P* < 0.01*
		PDZ 1 vs 3	*P* < 0.01*
		PDZ 2 vs 3	*P* < 0.05*
PC8 (3.0%)	*P* = 0.29	—	
PC9 (2.5%)	*P* = 0.79	—	
PC10 (2.3%)	*P* = 0.57	—	
T-test for single vs multi-celled organisms			
**PC (% Variance)**		***P*-value**	
**PC1 (24.5%)**		*P* < 0.01*	
PC2 (11.0%)		*P* = 0.85	
PC3 (8.1%)		*P* = 0.51	
**PC4 (5.0%)**		*P* < 0.01*	
PC5 (4.4%)		*P* = 0.19	
**PC6 (3.8%)**		*P* < 0.01*	
PC7 (3.5%)		*P* = 0.69	
PC8 (3.0%)		*P* = 0.72	
PC9 (2.5%)		*P* = 0.69	
PC10 (2.3%)		*P* = 0.70	

ANOVA was used for multiple comparisons between PDZ proteins with post hoc Tukey honest significant difference test. Welch's two tailed *t*-test was used for comparisons between single vs multi-celled organisms. *P* < 0.05 was considered statistically significant and are highlighted in bold. Dashed lines indicate where post hoc Tukey HSD tests were not used since ANOVA tests did not yield significant *P*-values.

#### AlphaFold Multimer Reveals Evolutionary Changes in the Conserved PDZ-CRIPT Binding Pocket

The mutations that proteins accumulate tend to result in structural changes, often with corresponding functional adaptations such as the acquisition of novel ligands. As PSD95 was co-opted in animals for a role at the synapse, the protein acquired associations with new proteins such as the NMDA and AMPA receptors that functionalized its role acquired in animals as a mediator of synaptic transmission. While the ESM-2 embeddings highlight evolutionary relationships, they do not assess functional conservation. To investigate how evolutionary changes impact PDZ binding, we used AlphaFold Multimer to conduct PDZ-CRIPT binding simulations with its reported accuracy for predicting multimeric interfaces (Evans et al. 2022). The results of the ESM-2 analysis reflected evolutionary divergence; however, our AlphaFold Multimer simulations demonstrated similar binding with the CRIPT ligand across diverse lineages, suggesting a functional conservation despite sequence variability. Among the PDZ3 PSD95 ligands, CRIPT is an ancient ligand present in the CO genomes (as well as AQ and HS). The CRIPT gene is present in Choanoflagellates (MB and SR) as well as ctenophores, but the ctenophore CRIPT does not contain a PDZ-binding consensus sequence and therefore was not included in this analysis.

We studied binding between PDZ3 and its endogenous synaptic ligand, CRIPT, using AlphaFold Multimer to derive a ligand interaction score (LIS score) (Evans et al. 2022; Kim et al. 2024). While predicted aligned error (pAE) is an inter-domain confidence metric, the derived LIS score calculation has increased sensitivity in the detection of protein-protein interactions with flexible and small interfaces, and focuses on areas of low pAE (Evans et al. 2022; Kim et al. 2024). A higher LIS score indicates a more confident prediction of a PDZ-ligand interaction.

We restricted our analysis to CO1, CO2, AQ, HS PDZs, and endogenous CRIPTs as these were the only sequences that contained the CRIPT consensus motif. The last ten amino acids of the CRIPT C-terminus are as follows: DTKNYKQTSV (*H. Sapiens*), DTSSYKQSVV (*C. owkczarzaki* CO1, CO2), DVKKYVQSTV (*A. queenslandica*), NFNRACVTA (*M. brevicollis*) and IDTKDLKQTN (*S. rosetta*). Each extant PDZ (CO1, CO2, AQ, HS) sequence was input to AlphaFold2 Multimer (Evans et al. 2022) with its CRIPT binding partner to predict their binding, using ten random seeds (Table 2, Table S2).

**Table 2.** AlphaFold multimer simulations LIS scores

Simulation (protein × ligand 10 aa)	LIS multimer
AQ PDZ3 × AQ CRIPT Average	**0.664** ± **0.004**
CO1 PDZ3 × CO CRIPT Average	**0.625** ± **0.003**
CO2 PDZ × CO CRIPT Average	**0.683** ± **0.005**
HS PDZ3 × HS CRIPT average	**0.679** ± **0.005**
Positive controls	
PDZ3 × VANGL2	0.65
PDZ3 × KALIRIN7	0.66
PDZ3 × CITRON	0.61
PDZ3 × SYNGAP	0.68
PDZ3 × HEXAPEPTIDE	0.75
Negative Controls	
HS PDZ 3 × HS NMDA	0.36
PDZ3 × GGGGGGGGGG	0.38
PDZ3 × AAAAAAAAAA	0.36
PDZ3 × TNF	0.67
PDZ3 × endozepine	0.48
PDZ3 × C-C motif chemokine 4 precursor	0.41

LIS scores derived from pAE interaction scores are shown for AF2-Multimer. Averages represent mean ± standard deviation for ten random seeds. CRIPT ligand is a known PDZ3 synaptic binding partner. NMDA receptor was the only literature-validated negative control; TNF, endozepine, and C-C motif chemokine 4 precursor are non-synaptic ligands.

**Fig. 5.**
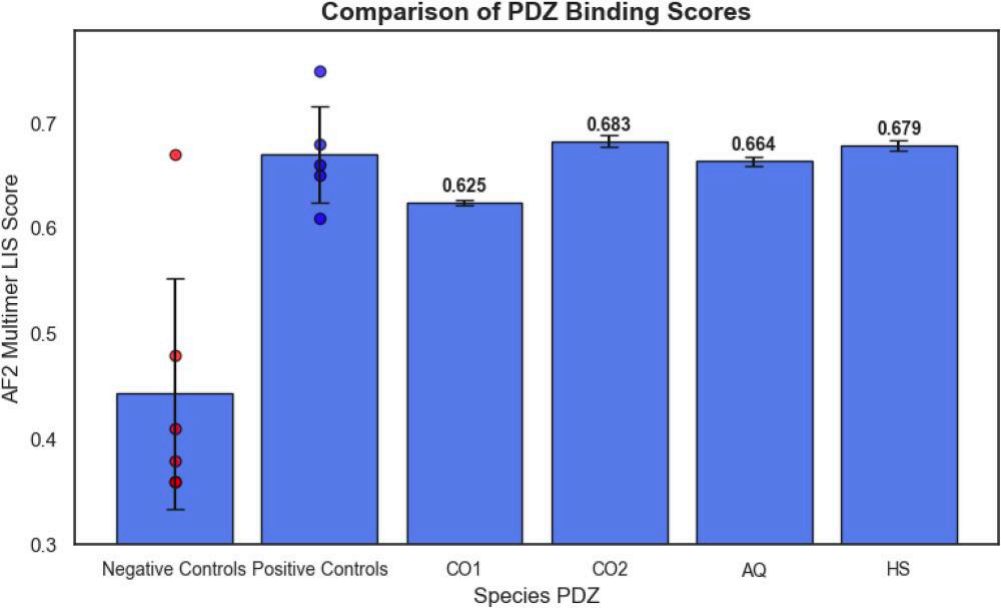
LIS score distribution sequences for human, CO2, CO1, and AQ with endogenous CRIPT ligand. Histogram of LIS scores and PDZ ligand (CO1, CO2, AQ, HS) are shown for ten random seeds. Error bars show standard deviation values.

All of the extant PDZ3 sequences—CO2, AQ, HS PDZ proteins—had similar predicted LIS scores with their endogenous CRIPT ligands. Interestingly, CO2 and HS bound their ligands with the highest LIS score while CO1 had the lowest LIS score (Fig. 5). The *pAE interaction* score (described by Bennett et al. 2023) and the pLDDT score—predicted local distance difference test (an amino acid-level confidence metric)—for each AF2-multimer prediction was also recorded (Table S3). The positive controls used were experimentally validated PDZ-binding partners and all displayed a LIS score >0.61 (Figure S2). Five positive controls were used to validate the methods that were known to bind experimentally with HS PDZ3: Syngap, Kalirin-7, Vangl-2, Citron, and a consensus hexapeptide peptide (Zhang et al. 1999; Penzes et al. 2001; Saro et al. 2007; Yoshioka et al. 2013). Non-synaptic ligands were also used as negative controls but did not have experimental validation; NMDA receptor was used as a literature-validated negative control that does not bind PDZ 3 (Kornau et al. 1995; Stricker et al. 1997; Cui et al. 2007; Wu and Tymianski 2018). Tumor necrosis factor (TNF), a non-synaptic ligand, had a high LIS binding score of 0.67; TNF interaction with PDZ has not been experimentally validated. pAE interaction and LIS score for all experimentally validated controls (five positive controls and one negative control) are shown in Supplementary Tables 2 and 3 and Figure S2, and free energy and potential energy scores of the AF2 predicted interaction of PDZ3 with the CRIPT ligand are shown in Table S5.

The single and multi-celled organisms all had LIS scores within the ranges of the positive controls tested (0.61 to 0.75), indicating potentially similar binding. Although the binding specificity of PDZ domains is determined by the interaction of highly conserved GLGF residues in human PDZs we found that in the absence of conserved GLGF residues among some single-celled PDZ domains, the endogenous CRIPT ligand still bound according to AF2-multimer. Prior studies have discussed the conservation of “GLGF” residues in the binding pocket of PDZ domains (Cho et al. 1992). Interestingly, single-celled organisms either with (CO2) or without (CO1) the highly conserved “GLGF” binding motif have similar CRIPT binding LIS scores (Fig. 5); although those with the “GLGF” binding motif have higher LIS scores than those without suggesting an early advantage of this motif.

### Sequence Conservation and Hydrophobicity Conservation Can Diverge

**Fig. 6.**
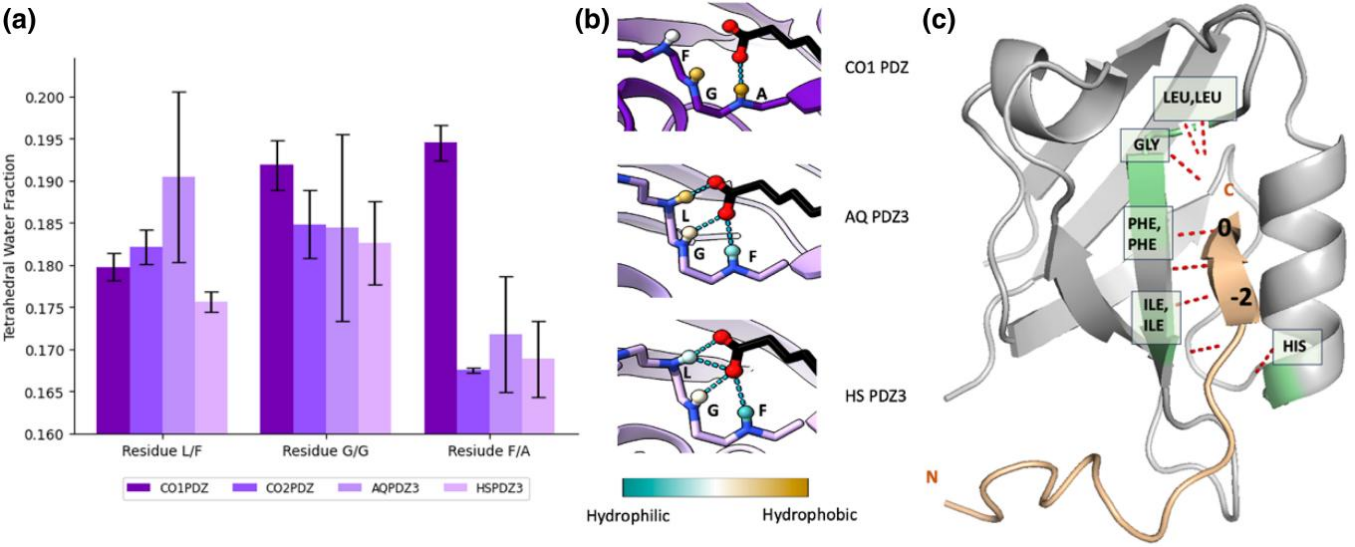
Comparison of average tetrahedral water fraction around hydrogen atoms for L, G, F residues and general conserved hydrogen bonds of PDZ protein with CRIPT a) histogram comparing average tetrahedral water fraction for L, G, F residues (corresponding, F, G, A residues for CO1) across three random seeds in molecular dynamics simulation. Error bars are standard deviation values. b) PDZ binding pocket interaction with the C-terminus of the CRIPT ligand; blue indicates hydrophilic atoms (low tetrahedrality) and yellow indicates hydrophobic atoms (high tetrahedrality). Scale bar represents values of 0.165 tetrahedral water fraction (blue) to 0.195 tetrahedral water fraction (yellow). c) The hydrogen bonding conservation was observed for the AF2-Multimer simulations. We evaluated CO1, CO2, HS, AQ PDZ3 with their endogenous CRIPT ligands. All binding simulations formed hydrogen bonds with the zero and −2 position of the ligand.

To better understand the contributing factors to binding conservation across single and multi-cellular organisms, we quantified the hydrophobicity in the PDZ ligand's binding pocket by measuring the local water structuring in molecular dynamics simulations (Lobo et al. 2025; Najafi et al. 2025). Water is seen to form more tetrahedral structures around small hydrophobic moieties, and fewer tetrahedral structures around hydrophilic moieties. We quantified the prevalence of these tetrahedral water angles with the water 3-body angle distribution (Monroe and Shell 2019; Jiao et al. 2022; Jiao et al. 2024). We measured water tetrahedrality around three key sites in the PDZ domain that interact with the ligand, namely the three backbone amide hydrogens in the final three residues of the famous “GLGF” motif (Songyang et al. 1997) (and the corresponding “SFGA” motif in CO1). These amide hydrogens can form hydrogen bonds with the charged carboxy group on the ligand's C-terminus; see Fig. 6b(iii).

Interestingly, we observed different hydrophobicity patterning of the binding pockets across divergent PDZ domains (Fig. 6). Human PDZ (HS PDZ3) had the lowest tetrahedral water fraction, and consequently the highest hydrophilicity, at the hydrogen bond donors in its binding pocket, while CO1 had the highest tetrahedral water fraction and lowest hydrophilicity. HS PDZ3's increased hydrophilicity may explain why it was predicted to have many hydrogen bonds and a high LIS score with CRIPT according to AF2-multimer, while CO1's lower hydrophilicity corresponds to fewer hydrogen bonds and a lower LIS score with its CRIPT. Most notably, water tetrahedrality was significantly higher for CO1 around its Ala residue (corresponding to Phe residue in humans) than the other three proteins (Fig. 6a). The decreased hydrophilicity may make it more difficult for the CO1 PDZ to interact with the charged C terminus of CRIPT; CO1's three amide hydrogens do not appear to point directly toward the pocket to optimize hydrogen bonding like they do in the three “GLGF”-containing PDZs (Fig. 6b).

“GLGF” may thus be a functional adaptation that allows CO2, AQ, and HS to interact more strongly with their ligands, i.e. higher LIS scores, while proteins without GLGF may have evolved different solutions to accommodate ligands. Thus, we observe that proteins with “GLGF” appear to have distinct sequence conservation and physiochemical property conservation that serves to retain consistent binding profiles with the endogenous CRIPT ligand. Proteins without “GLGF” are still able to bind the CRIPT ligand, but the differing hydrophobicity profiles suggest functional flexibility as differing hydrophobicity does not necessarily disrupt ligand binding. The AF2-Multimer predictions suggest that all four of these PDZs will interact with their ligand despite hydrophobicity differences, and this demonstrates functional persistence despite sequence and structural divergence.

#### Conservation of Hydrogen Bonds Between PDZ and CRIPT

While sequence and hydrophobicity diverged within a conserved binding domain, remarkably hydrogen bond interactions at consensus positions 0 and −2 of PSD-95 CRIPT ligand were conserved over evolutionary distances that spanned protista to animals. These hydrogen bonds are conserved across *H. sapiens*, the sponge *A. queenslandica*, and the unicellular *C. owczarzaki*. We posit that the hydrogen bond interactions at specific ligand positions, and not necessarily conserved residue identities at the sequence level, may have enabled new ligand partners for PSD-95.

Hydrogen bonding was characterized from single-celled organisms to multi-celled organisms. Simulations for *H. sapiens, A. queenslandica*, and *C. owczarzaki* (CO1 and CO2) were evaluated with endogenous CRIPT ligands in AlphaFold2 Multimer analyzed for hydrogen bond conservation. All simulations had conserved hydrogen bonding at the zero and −2 position of the ligand. The general consensus hydrogen bonds are displayed at the zero and −2 position (Fig. 6c). The H-bond pattern does not seem to be sensitive to the random seed. The PDZ domains of different species have different binding sequences, and thus there was variation regarding which residues form these bonds and suggest the importance of hydrogen bonding in long time scale conservation in maintaining a protein-protein interaction that supersede sequence changes. Characterization of these bonds and their variations can be seen in Table 3. As far as we are aware, hydrogen bonds formed between the PDZ domain and its ligand have not been described previously.

**Table 3:** All hydrogen bonds formed between PDZ domain and CRIPT ligand for AF2-Multimer

Ligand sites	CO1PDZ3 × COCRIPT	CO2PDZ3 × COCRIPT	HSPDZ3 × HS CRIPT	AQPDZ3 × AQCRIPT
0 site	A16bb-bb	L13bb-bb	L14bb-bb	L12bb-bb
	A16bb-bb	L13bb-bb	L14bb-bb	L12bb-bb
		G14bb-bb	G15bb-bb	G13bb-bb
		F15bb-bb	F16bb-bb	F14bb-bb
		F15bb-bb	F16bb-bb	F14bb-bb
-1 site				
-2 site	V18bb-bb	I17bb-bb	I18bb-bb	I16bb-bb
	V18bb-bb	H63sc-sc	H63sc-sc	I16bb-bb
		I17bb-bb	I18bb-bb	
-3 site	T17sc-sc			N15sc-sc
				N15sc-sc
-4 site		G19bb-bb	E64sc-sc	
		Q23sc-bb		
		Q23sc-bb		
-5 site				

bb-bb refers to backbone-backbone hydrogen bonds and sc-sc refers to side chain-side chain hydrogen bonds formed between residues on the PDZ domain and CRIPT ligand.

## Discussion

Although DLG4 is broadly distributed in the animal kingdom, its presence in basal animals such as the sponge that lacks synapses suggests its ancestral functional roles were unrelated to the synapse. Choanoflagellates have the same DLG4 domain architecture as found in the animal kingdom. Each of their three PDZ domains cluster with PDZ one, two, or three in animals, although we previously reported that in *M. brevicollis* some residues that classify PDZ domains within their precise order were lost (Sakarya et al. 2010). A yeast two-hybrid screen in *M. brevicollis* revealed ligands with carboxy-terminal PDZ-binding sequences and among the known genes most were proteases (Sakarya et al. 2010) suggesting that one function of an ancient DLG4 complex served as a mediator of protein degradation. The very successful tri-PDZ architecture originated with a PDZ3-like domain that is ancestral to PDZ1 and PDZ2 (Fig. 2). Our findings support previous work suggesting that within the DLG superclass, the MAGUK core architecture was further elaborated in the Choanoflagellata lineage, where duplication of the most C-terminal PDZ domain gave rise to the DLG, ZO and CARMA subfamilies (Fig. 1). We also identified DLG4 homologs in Ctenophores, where they had not been previously documented, expanding the known distribution of this gene family and suggesting that the DLG4 complex may have supported early forms of neural organization in the animal stem group (Fig. 1).

We extended the sequence-based analysis to explore the structural relationships of PDZ proteins and their potential connection to binding function. Using ESM, we investigated how residue conservation relates to relationships observed in the phylogeny and protein structure. Our analyses demonstrated distinct PDZ 1, 2, 3, groupings according to their ESM, consistent with clade formation in the phylogeny. Interestingly, the PCA of the ESM embeddings further separated single and multi-cellular organisms. For example, while HS VAM-1 and CO1, proteins that share the PDZ-SH3-GK architecture, reside in the same phylogenetic clade, they were separated in the PCA plot of the ESM. A similar observation was made with CO2 and HS PDZ3. These findings suggested other contributing factors to PDZ function beyond residue conservation.

Additional analyses corroborated that shared sequence similarity was not a predictor of binding. This was evident in the AlphaFold Multimer binding simulations with CO1, CO2, HS, and AQ with their endogenous CRIPT ligands, which shared similar LIS binding scores suggesting that all proteins retained binding capability. Interestingly, this similarity in binding did not appear to be due to shared properties in the binding pocket, nor “GLGF” sequence conservation which is absent in CO1, despite the prevalent view that the “GLGF” is characteristic of all PDZ domains. The molecular dynamics simulation demonstrated that the proteins that shared “GLGF” sequence similarity (CO2 and HS PDZ3) had similarities in hydrophobicity patterning of the binding pocket, while those without “GLGF” (CO1) had different hydrophobicity patterns, but this did not significantly alter binding function. Instead, our results highlight the importance of positional hydrogen binding conservation. We found conserved PDZ-ligand hydrogen bonding at the 0 and −2 position of the ligand in both single and multi-cellular organisms. Our results suggest that evolutionary pressures may have preserved ligand and binding pocket features through hydrogen bonding as a determinant for PDZ- CRIPT interactions. The role of inherently quantum mechanical hydrogen bonds in an early exploration of protein-protein interaction space opens the possibility of a quantum mechanical-based optimization mode (Li et al. 2011) potentially capable of capturing a broader range of ligands to enhance fitness, and thereby speed evolution while waiting for the mutations that can secure specific ligand binding. In this case, an ancient transition appeared to involve an interaction mediated in part by hydrogen bonding to the more specific and stronger GLGF sequence.

## Methods

### Sequence Analysis

#### Database Search for DLG-4 Like Homologs

An initial search for human DLG-4 homologs in premetazoan lineages was obtained by performing BLAST searches against NCBI (National Center for Biotechnology Information). Premetazoan species searched for homologs, including two representatives from Choanoflagellata, *M. brevicollis* (MB), and *S. rosetta* (SR), and one representative from Filasterea, *C. owczarzaki* (CO). The complete protein architecture of top blast hits was assessed using NCBI CD-SEARCH. DLG-like homologs were retained if sequences had the MAGUK protein architecture, with at least one PDZ, SH3, and Guk domains.

#### Phylogenetic Analysis of sh3+guk

For all metazoan MAGUK genes, we downloaded the protein alignments of sh3+guk genes from de Mendoza et al. (2010). To this alignment, we added the sh3+guk genes of DLG-like homologs found in *M. brevicollis* (MB), *S. rosetta* (SR), one representative from Filasterea, *C. owczarzaki* (CO), and two Ctenophore species *B. infundibulum* and *M. leidyi*. We used MAFFT to realign all metazoan and premetazoan sh3+guk genes. Maximum likelihood phylogenetic trees were estimated using IQ-TREE2 using the model of evolution WAG+*Γ* estimated by Mendoza et al. (de Mendoza et al. 2010), and statistical support was obtained using the ultrafast bootstrap parameter (*n* = 1000) in IQ-TREE2. The treefile was viewed in the Interactive Tree of Life (iTOL). We rooted the sh3+guk gene tree in the branch between DLG-like+DLG and MPP clades.

#### Phylogenetic Analysis of PDZ Domains

We used a custom R script to extract PDZ domains from each DLG-like in premetazoan species and DLG4 genes in metazoan species. We extracted all N-terminus PDZ domains before the start of the sh3+guk domain for each gene. To extract the PDZ domains, we extended the boundaries of predicted domain boundaries generated by NCBI CD-SEARCH by ten amino acids in the front of each PDZ domain and ten amino acids at the end of each PDZ domain. We used MAFFT to align the protein sequences of PDZ domains. Maximum likelihood phylogenetic trees were estimated using IQ-TREE2 (Minh et al. 2020) using the best-fit model of evolution predicted by ModelFinder implemented by IQ-TREE2. Statistical support was obtained using the ultrafast bootstrap parameter in IQ-TREE2. The treefile was viewed in the iTOL. We used the midpoint root function in iTOL (Letunic and Bork 2021) to midpoint root the PDZ domain phylogenetic tree.

#### Ancestral Sequence Reconstruction of PDZ Domains Within DLG 1-4 and DLG-like Clades

The ancestral PDZ sequences were inferred on the basis of the PDZ phylogenetic tree of the extant amino acid sequences. Ancestral sequences at internal nodes in the PDZ phylogenetic tree were inferred using IQ-TREE. Posterior amino acid probabilities at each amino acid site were calculated using LG+G4, given the ML model tree generated by IQ-TREE with a minimum threshold of 0.5. To trace the evolutionary history of the three PDZ domains (PDZ1, PDZ2, and PDZ3) of the DLG 1 to 4 subfamily, we targeted three ancestral domains encoding the ancestral PDZs of PDZ1 and PDZ2 (Node 11; Fig. 2); of PDZ3 and PDZ1-2 (Node 6; Fig. 2); of *C. owczarzaki* C01 PDZ3 and PDZ1-2-3 (Node 32; Fig. 2). The posterior probabilities of the amino acid residues in the three reconstructed PDZ domains were high, on average >0.8.

### Structural Analysis

https://github.com/samlobe/synapse-evolution

#### Evolutionary Scale Modeling

We extracted the 5120 embeddings for each PDZ sequence from the pretrained esm2_t48_15B_UR50D model (15B parameters and 48 layers) using this command: python extract.py esm2_t48_15B_UR50D PDZs.fasta output_dir –include mean

where PDZs.fasta contains each PDZ sequence and “–include mean” averages across all the amino acids in the sequence. The extract.py can be found here: (https://github.com/facebookresearch/esm, Rives et al. (2021)). Then we performed PCA, i.e. unsupervised learning, on the embeddings. ANOVA tests with post hoc Tukey honest significant difference for pairwise comparisons were performed for differences between PDZ domains (PDZ1 vs PDZ2 vs PDZ3). Welch's two tailed *t*-tests were performed to test for statistical significance between single vs multi-celled organisms. A *P*-value <0.05 was considered statistically significant.

#### Protein Structure Prediction

We used the LocalColabFold for structure prediction, which implements ColabFold locally (Mirdita et al. 2022). ColabFold is an implementation of AlphaFold2 (Jumper et al. 2021) that uses the faster MMseqs2 (Steinegger and Söding 2017) method to create the MSA for structure prediction. For each structure prediction, we input the full PDZ sequence followed by a chain break and the last ten residues of the ligand. All the AlphaFold Multimer settings were set to their defaults except we reduced the number of recycles to five per the protocol in Kim et al. (Kim et al. 2024). We derived the LIS score from the top ranked structure's pAE matrix as described by Kim et al. Specifically, we looked at the interchain elements of the pAE matrix with values below 12, scaled them to a 0-1 scale (where 0 represents pAE = 12 and 1 represents pAE = 0), and averaged those values to get the LIS score.

#### Free Energy and Total Energy Analysis

The intermolecular potential energy between each PDZ protein and its ligand (Table S5) was measured using OpenMM and the AMBER14 force field with OBC2 generalized Born implicit solvent model (amber14-all.xml and implicit/obc2.xml). We referred to the Computing Interaction Energies cookbook from OpenMM. Energies were computed after minimizing. Structures were minimized three independent times with the L-BFGS algorithm and we report the average potential energy.

PyRosetta and the ref2015_cart all-atom were used to measure the binding free energy (ΔG_bind) between PDZ protein and ligand (Table S5). AlphaFold 2 was used to generate PDB structures, and structures were preprocessed by relaxing with the FastRelax protocol. Then, InterfaceAnalyzerMover was applied to the PDZ-ligand interface to find ΔG_bind. All calculations were run using PyRosetta.

#### Hydrophobicity Analysis

We modeled the water structure around each PDZ protein by simulating water-protein atoms in a MD simulation with the a99SBdisp force field (Robustelli et al. 2018) and then measured the water triplet distribution of PDZ's hydration waters. We took the AlphaFold2-predicted structure of the PDZ monomer as the starting structure, capped the N- and C-terminus with ACE- and NME- caps, respectively, solvated the protein with water (a99Sbdisp water, a modified TIP4P-D model), and added Na+ or Cl– ions to neutralize the charge. Using OpenMM (Eastman et al. 2017), the energy is minimized with the L-BFGS algorithm (Liu and Nocedal 1989), the system is equilibrated for 100 ps, and then the system is simulated for 5 ns in the NPT ensemble (*T* = 300 K and *P* = 1 atm). Nonbonded interactions were treated using the PME method (Darden et al. 1993) with a 1.0 nm nonbonded cutoff. Bonds with hydrogen atoms were constrained with the SHAKE algorithm (Ryckaert et al., 1977). Temperature was controlled using a Langevin integrator with a middle scheme, set with a friction coefficient of 2 ps-1 and an integration timestep of 4 fs (Schlick 2010). Pressure was regulated using a Monte Carlo Barostat (Allen and Tildesley 2017), updating every 25 simulation steps.

Water structure is quantified in each picosecond of the 5-ns trajectory. Specifically, we compute the water triplet distribution (also called the water three-body angle distribution) as described by Monroe and Shell (2019), which involves looping through each hydration water in each frame of the MD simulation and measuring the angles it makes with neighboring water's oxygens. A hydration water is any water whose oxygen is within 4.25 Å of a heavy atom in the amino acid. The water triplet distribution was computed for the three hydrogen bond donors in the PDZ, as shown in Fig. 6a, and the tetrahedral water fraction was calculated by integrating the water triplet distribution from 100 to 120°. This MD simulation was performed for each PDZ three times, totaling 15 ns of simulation time per PDZ, and the average tetrahedrality from these three simulations was reported.

#### Hydrogen Bond Analysis

We counted the intermolecular hydrogen bonds between PDZ domains and ligands whose structures were predicted using AlphaFold2. We define a hydrogen bond as having a donor-acceptor cutoff of 3.7 Å and an angle cutoff of 120° (donor-hydrogen-acceptor). These hydrogen bonds were measured using MDAnalysis (Michaud-Agrawal et al. 2011). The hydrogen bonds shown in Fig. 6b and c were found using the “hbond” command in ChimeraX.

## Supplementary Material

msaf309_Supplementary_Data

## Data Availability

All datasets used in this manuscript are publicly available on NCBI, with accession numbers provided in the Supplementary files.
