# Supplementary material for: Evolution of the Tri-PDZ Domain in PSD95 (DLG-4 Gene)
Source: Mol Biol Evol. 2025 Dec 17;42(12):msaf309. doi: 10.1093/molbev/msaf309 (PMC12709283; doi:10.1093/molbev/msaf309)
Supplement: msaf309_Supplementary_Data [file msaf309_supplementary_data.pdf]

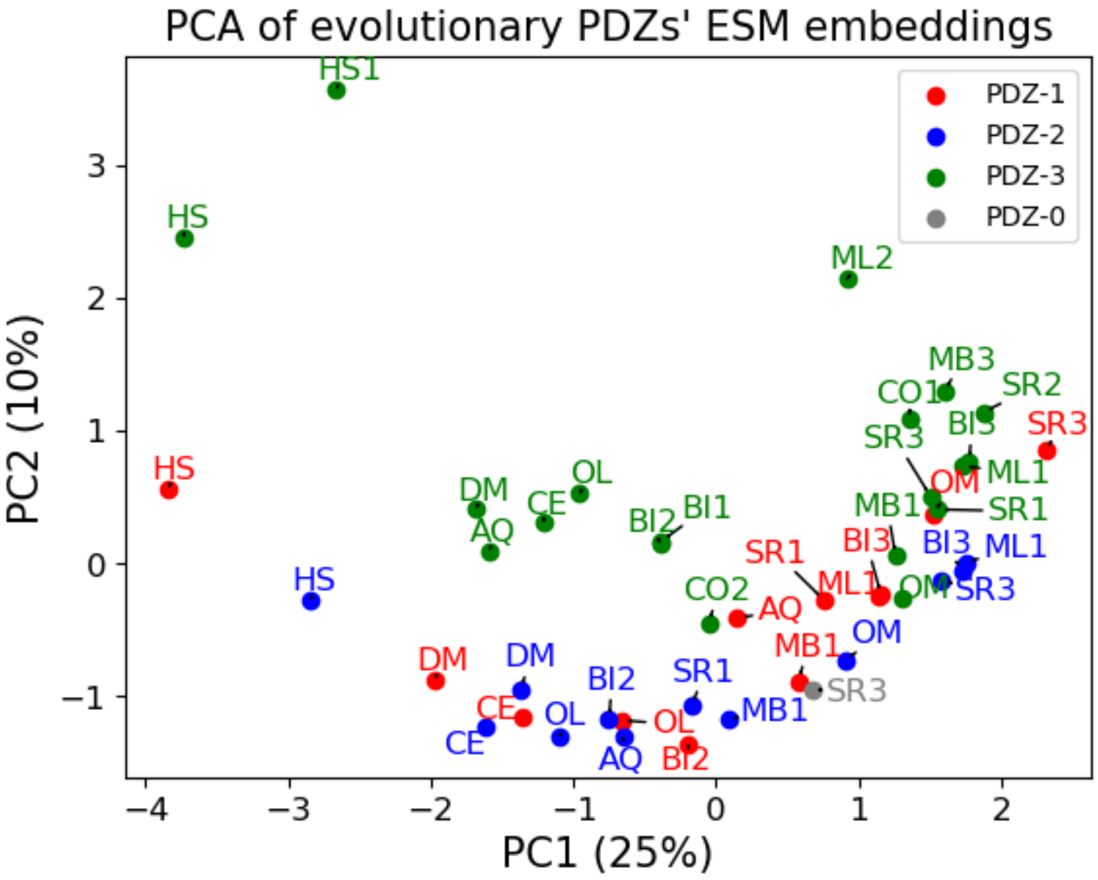

Supplementary Figure 1. Annotated PCA of ESM for all genes in phylogeny. Naming conventions can be found in Supplementary Table 1.

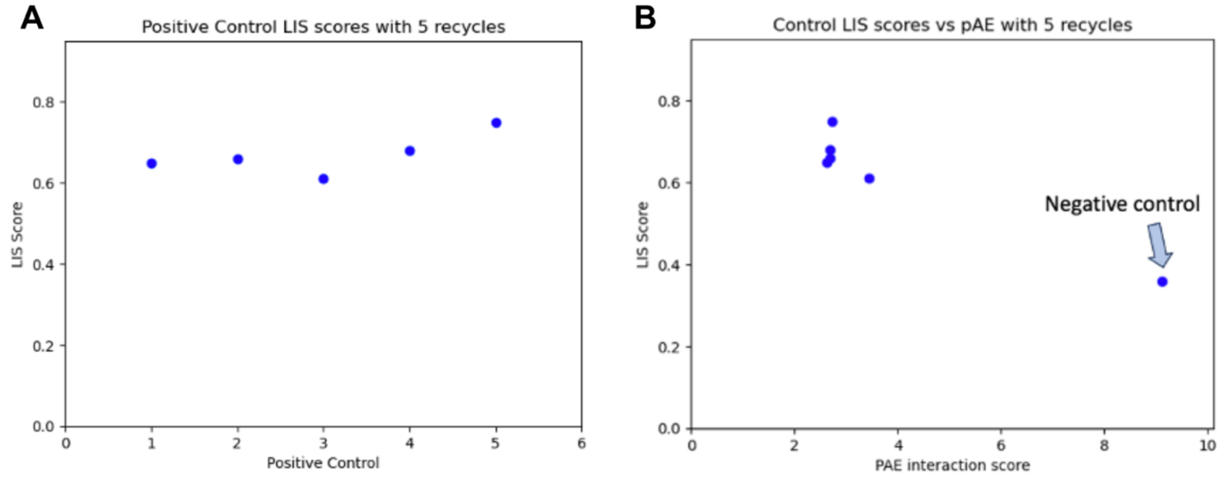

Supplementary Figure 2. A) LIS scores for the 5 positive controls. B). LIS vs pAE for 5 positive controls and 1 negative control (NMDA receptor) that have been experimentally validated. PAE interaction scores were calculated in reference to methodologies described by Bennett et al[23].

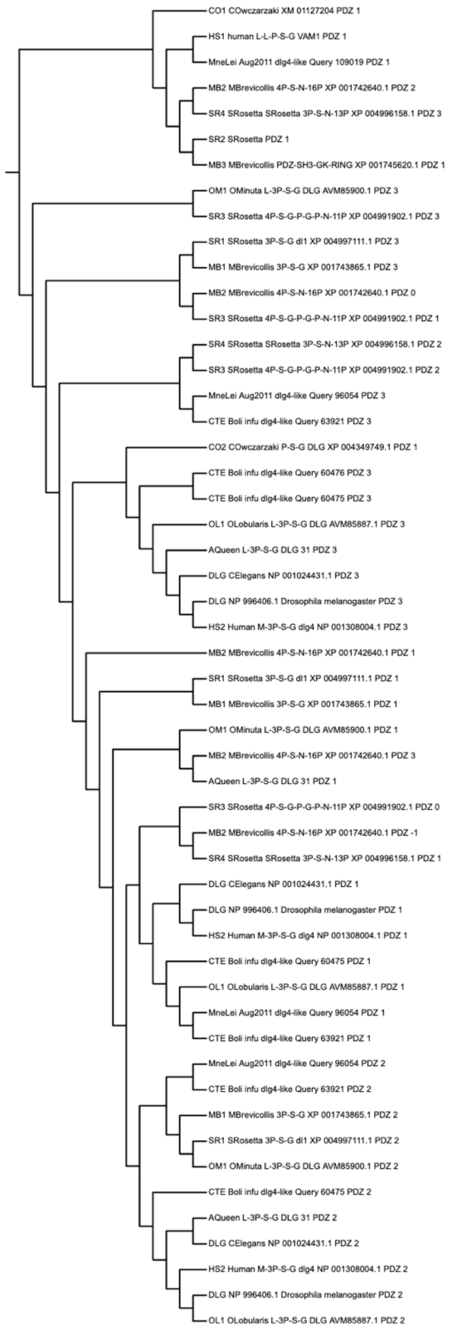

Supplementary Figure 3: Phylogeny of PDZ domain sequences of *H. sapiens* DLG4 gene and DLG4-like homologs in single-celled relatives including PDZ domains from Sh3+NK genes. The topology and branch lengths were obtained by maximum likelihood analysis using IQ-TREE2. The tree is midpoint rooted. In addition to all N-terminus PDZs from genes with MAGUK sh3+guk architecture, all N-terminus PDDZ from MB2 and SR4 genes that possess the Sh3+NK architecture are also included in this tree, totaling 52 PDZ domains.

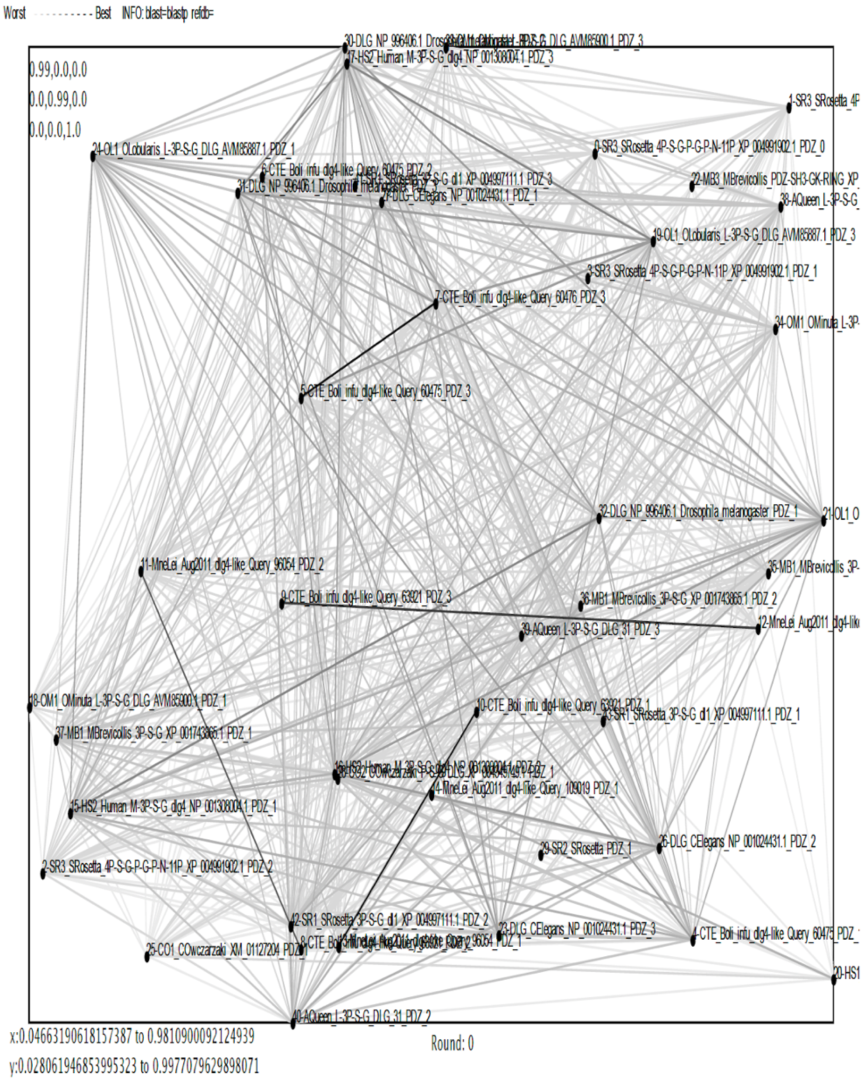

Supplementary Figure 4: CLANS clustering of PDZ sequences. CLANS clustering of PDZ sequences shows that PDZ domains lacking key binding residues (including *C. owczarzaki* CO1) cluster with PDZ domains from single-celled organisms that also lack key binding residues. PDZ domains containing key binding residues cluster (including *C. owczarzaki* CO2) form distinct clusters with metazoan PDZs that also have key binding residues.

Sequence Name, Architecture, Accession Number	Annotation for the PDZ tree and ESM embeddings
SR3_SRosetta_4P-S-G-P-G-P-N-11P_XP_004991902.1_PDZ_0	SR3 *S. rosetta* PDZ 0
SR3_SRosetta_4P-S-G-P-G-P-N-11P_XP_004991902.1_PDZ_3	SR3 *S. rosetta* PDZ 3
SR3_SRosetta_4P-S-G-P-G-P-N-11P_XP_004991902.1_PDZ_2	SR3 *S. rosetta* PDZ 2
SR3_SRosetta_4P-S-G-P-G-P-N-11P_XP_004991902.1_PDZ_1	SR3 *S. rosetta* PDZ 1
CTE_Boli_infu_3P-S-G_Query_60475_GCA_026151205.1_PDZ_1	BI2 *B. infundibulum* PDZ 1
CTE_Boli_infu_3P-S-G_Query_60475_GCA_026151205.1__PDZ_3	BI2 *B. infundibulum* PDZ 3
CTE_Boli_infu_3P-S-G_Query_60475_GCA_026151205.1_PDZ_2	BI2 *B. infundibulum* PDZ 2
CTE_Boli_infu_P-S-G_Query_60476_GCA_026151205.1_PDZ_3	BI1 *B. infundibulum* PDZ 3
CTE_Boli_infu_3P-S-G_Query_63921_GCA_026151205.1_PDZ_2	BI3 *B. infundibulum* PDZ 2
CTE_Boli_infu_3P-S-G_Query_63921_GCA_026151205.1_PDZ_3	BI3 *B. infundibulum* PDZ 3
CTE_Boli_infu_3P-S-G_Query_63921_GCA_026151205.1_PDZ_1	BI3 *B. infundibulum* PDZ 1
MneLei_Aug2011_3P-S-G_Query_96054_GCA_000226015.1_PDZ_2	ML1 *M. leidyu* PDZ 2
MneLei_Aug2011_3P-S-G_Query_96054_GCA_000226015.1_PDZ_3	ML1 *M. leidyu* PDZ 3
MneLei_Aug2011_3P-S-G_Query_96054_GCA_000226015.1_PDZ_1	ML1 *M. leidyu* PDZ 1
MneLei_Aug2011_P-S-G_Query_109019_GCA_000226015.1_PDZ_1	ML2 *M. leidyu* PDZ 3
HS2_Human_M-3P-S-G_dlg4_NP_001308004.1_PDZ_1	HS *H. sapiens* PDZ 1
HS2_Human_M-3P-S-G_dlg4_NP_001308004.1_PDZ_2	HS *H. sapiens* PDZ 2
HS2_Human_M-3P-S-G_dlg4_NP_001308004.1_PDZ_3	HS *H. sapiens* PDZ 3
OM1_OMinuta_L-3P-S-G_DLG_AVM85900.1_PDZ_1	OM *O. minuta* PDZ 1
OL1_OLobularis_L-3P-S-G_DLG_AVM85887.1_PDZ_3	OL *O. lobularis* PDZ 3
HS1_human_L-L-P-S-G_VAM1_AAD45919.2_PDZ_1	HS1 *H. sapiens* PDZ 3

OL1_OLobularis_L-3P-S-G_DLG_AVM85887.1_PDZ_2	OL *O. lobularis* PDZ 2
MB3_MBrevicollis_PDZ-SH3-GK-RING_XP_001745620.1_PDZ_1	MB3 *M. brevicollis* PDZ 3
OL1_OLobularis_L-3P-S-G_DLG_AVM85887.1_PDZ_1	OL *O. lobularis* PDZ 1
CO1_COwczarzaki_P-S-G_XM_01127204_PDZ_1	CO1 *C. owczarzaki* PDZ 3
DLG_CElegans_ L-3P-S-G-N_NP_001024431.1_PDZ_3	CE *C. elegans* PDZ 3
DLG_CElegans_ L-3P-S-G-N_NP_001024431.1_PDZ_2	CE *C. elegans* PDZ 2
DLG_CElegans_ L-3P-S-G-N_NP_001024431.1_PDZ_1	CE *C. elegans* PDZ 1
CO2_COwczarzaki_P-S-G_DLG_XP_004349749.1_PDZ_1	CO2 *C. owczarzaki* PDZ 3
SR2_SRosetta_L-P-S-G_XP_004989218.1_PDZ_1	SR2 *S. rosetta* PDZ 3
DLG_ L-3P-S-G _NP_996406.1_Drosophila_melanogaster_PDZ_2	DM *D. melanogaster* PDZ 2
DLG_ L-3P-S-G _NP_996406.1_Drosophila_melanogaster_PDZ_3	DM *D. melanogaster* PDZ 3
DLG_ L-3P-S-G _NP_996406.1_Drosophila_melanogaster_PDZ_1	DM *D. melanogaster* PDZ 1
OM1_OMinuta_L-3P-S-G_DLG_AVM85900.1_PDZ_3	OM *O. minuta* PDZ 3
OM1_OMinuta_L-3P-S-G_DLG_AVM85900.1_PDZ_2	OM *O. minuta* PDZ 2
MB1_MBrevicollis_3P-S-G_XP_001743865.1_PDZ_3	MB1 *M. brevicollis* PDZ 3
MB1_MBrevicollis_3P-S-G_XP_001743865.1_PDZ_2	MB1 *M. brevicollis* PDZ 2
MB1_MBrevicollis_3P-S-G_XP_001743865.1_PDZ_1	MB1 *M. brevicollis* PDZ 1
AQueen_L-3P-S-G_DLG_31_PDZ_1	AQ *A. queenslandica* PDZ 1
AQueen_L-3P-S-G_DLG_31_PDZ_3	AQ *A. queenslandica* PDZ 3
AQueen_L-3P-S-G_DLG_31_PDZ_2	AQ *A. queenslandica* PDZ 2
SR1_SRosetta_3P-S-G_dl1_XP_004997111.1_PDZ_3	SR1 *S. rosetta* PDZ 3
SR1_SRosetta_3P-S-G_dl1_XP_004997111.1_PDZ_2	SR1 *S. rosetta* PDZ 2
SR1_SRosetta_3P-S-G_dl1_XP_004997111.1_PDZ_1	SR1 *S. rosetta* PDZ 1

Supplementary Table 1. Naming conventions and accession numbers of genes used in PDZ phylogeny and ESM analysis. The sequence name lists the species abbreviation, the

architecture of the gene (P: PDZ, S: SH3, G: GK domains), the accession number, and the corresponding PDZ domain. PDZ3 was labeled as the C-terminal PDZ adjacent to the SH3 domain.

Simulation (Protein x Ligand 10 aa)	LIS Multimer
AQ PDZ3 x AQ CRIPT seed 10	0.661
AQ PDZ3 x AQ CRIPT seed 11	0.658
AQ PDZ3 x AQ CRIPT seed 12	0.668
AQ PDZ3 x AQ CRIPT seed 15	0.661
AQ PDZ3 x AQ CRIPT seed 25	0.670
AQ PDZ3 x AQ CRIPT seed 32	0.662
AQ PDZ3 x AQ CRIPT seed 48	0.667
AQ PDZ3 x AQ CRIPT seed 58	0.661
AQ PDZ3 x AQ CRIPT seed 71	0.670
AQ PDZ3 x AQ CRIPT seed 88	0.659
CO1 PDZ3 x CO CRIPT seed 10	0.625
CO1 PDZ3 x CO CRIPT seed 11	0.625
CO1 PDZ3 x CO CRIPT seed 12	0.622
CO1 PDZ3 x CO CRIPT seed 15	0.623
CO1 PDZ3 x CO CRIPT seed 25	0.626
CO1 PDZ3 x CO CRIPT seed 32	0.623
CO1 PDZ3 x CO CRIPT seed 48	0.623
CO1 PDZ3 x CO CRIPT seed 58	0.625
CO1 PDZ3 x CO CRIPT seed 71	0.632
CO1 PDZ3 x CO CRIPT seed 88	0.625
CO2 PDZ3 x CO CRIPT seed 10	0.685
CO2 PDZ3 x CO CRIPT seed 11	0.676
CO2 PDZ3 x CO CRIPT seed 12	0.687
CO2 PDZ3 x CO CRIPT seed 15	0.687
CO2 PDZ3 x CO CRIPT seed 25	0.685
CO2 PDZ3 x CO CRIPT seed 32	0.682
CO2 PDZ3 x CO CRIPT seed 48	0.679
CO2 PDZ3 x CO CRIPT seed 58	0.673
CO2 PDZ3 x CO CRIPT seed 71	0.693
CO2 PDZ3 x CO CRIPT seed 88	0.684
HS PDZ3 x HS CRIPT seed 10	0.684
HS PDZ3 x HS CRIPT seed 11	0.675

HS PDZ3 x HS CRIPT seed 12	0.683
HS PDZ3 x HS CRIPT seed 15	0.675
HS PDZ3 x HS CRIPT seed 25	0.674
HS PDZ3 x HS CRIPT seed 32	0.685
HS PDZ3 x HS CRIPT seed 48	0.686
HS PDZ3 x HS CRIPT seed 58	0.679
HS PDZ3 x HS CRIPT seed 71	0.677
HS PDZ3 x HS CRIPT seed 88	0.673

Supplementary Table 2. AlphaFold Multimer Simulations for 10 Random seeds. LIS scores derived from pAE_interaction scores are shown forAF2 Multimer. Averages represent mean ± standard deviation. CRIPT ligand is a known PDZ3 synaptic binding partner.

Simulation (Protein x Ligand 10 aa)	pLDDT	pAE
AQ PDZ3 x AQ CRIPT seed 10	96.61	2.16
AQ PDZ3 x AQ CRIPT seed 11	96.45	2.16
AQ PDZ3 x AQ CRIPT seed 12	96.0	2.16
AQ PDZ3 x AQ CRIPT seed 15	96.5	2.16
AQ PDZ3 x AQ CRIPT seed 25	96.18	2.16
AQ PDZ3 x AQ CRIPT seed 32	96.61	2.16
AQ PDZ3 x AQ CRIPT seed 48	97.81	2.16
AQ PDZ3 x AQ CRIPT seed 58	96.58	2.16
AQ PDZ3 x AQ CRIPT seed 71	96.02	2.16
AQ PDZ3 x AQ CRIPT seed 88	96.42	2.16
CO1 PDZ3 x CO CRIPT seed 10	95.33	2.25
CO1 PDZ3 x CO CRIPT seed 11	95.42	2.25
CO1 PDZ3 x CO CRIPT seed 12	95.47	2.25
CO1 PDZ3 x CO CRIPT seed 15	95.38	2.25
CO1 PDZ3 x CO CRIPT seed 25	95.55	2.25
CO1 PDZ3 x CO CRIPT seed 32	95.55	2.25
CO1 PDZ3 x CO CRIPT seed 48	95.42	2.25
CO1 PDZ3 x CO CRIPT seed 58	95.45	2.25
CO1 PDZ3 x CO CRIPT seed 71	95.89	2.25
CO1 PDZ3 x CO CRIPT seed 88	95.61	2.25
CO2 PDZ3 x CO CRIPT seed 10	96.33	2.34
CO2 PDZ3 x CO CRIPT seed 11	96.22	2.34
CO2 PDZ3 x CO CRIPT seed 12	96.44	2.34
CO2 PDZ3 x CO CRIPT seed 15	96.36	2.34
CO2 PDZ3 x CO CRIPT seed 25	96.22	2.34
CO2 PDZ3 x CO CRIPT seed 32	96.26	2.34
CO2 PDZ3 x CO CRIPT seed 48	96.11	2.34
CO2 PDZ3 x CO CRIPT seed 58	96.24	2.34
CO2 PDZ3 x CO CRIPT seed 71	96.80	2.34
CO2 PDZ3 x CO CRIPT seed 88	96.28	2.34
HS PDZ3 x HS CRIPT seed 10	96.98	2.18
HS PDZ3 x HS CRIPT seed 11	96.41	2.18

HS PDZ3 x HS CRIPT seed 12	96.94	2.18
HS PDZ3 x HS CRIPT seed 15	96.75	2.18
HS PDZ3 x HS CRIPT seed 25	96.64	2.18
HS PDZ3 x HS CRIPT seed 32	96.89	2.18
HS PDZ3 x HS CRIPT seed 48	97.12	2.18
HS PDZ3 x HS CRIPT seed 58	96.56	2.18
HS PDZ3 x HS CRIPT seed 71	96.85	2.18
HS PDZ3 x HS CRIPT seed 88	96.58	2.18
**Positive Control**		
PDZ3 x VANGL2	96.28	2.62
PDZ3 x KALIRIN7	95.76	2.68
PDZ3 x CITRON	94.61	3.43
PDZ3 x SYNGAP	97.11	2.68
PDZ3 x HEXAPEPTIDE	97.36	2.73
**Negative Control**		
HS PDZ 3 x HS NMDA	59.72	9.13
PDZ3 x GGGGGGGGGG	49.49	11.89
PDZ3 x AAAAAAAAAA	41.80	13.30
PDZ3 x TNF	94.97	3.57
PDZ3 x endozepine	79.70	5.96
PDZ3 x C-C motif chemokine 4 precursor	63.42	8.51

Supplementary Table 3. Results of AF2 Multimer binding simulations of the PDZ protein and 10 amino acids of the CRIPT ligand. Binding is defined as a pLDDT score greater than 70 (https://alphafold.ebi.ac.uk/faq). pAE scores refer to the pAE interaction scores reported by Bennett et al [23]. The number of recycles does not seem to cause differences in scores.

**Sequence Name, Architecture, Accession Number**	**Annotation for sh3+ NK genes and their corresponding PDZ domains**
MB2_MBrevicollis_4P-S-N-16P_XP_001742640.1_PDZ_3	MB2 *M.brevicollis* PDZ 3
MB2_MBrevicollis_4P-S-N-16P_XP_001742640.1_PDZ_2	MB2 *M.brevicollis* PDZ 2
MB2_MBrevicollis_4P-S-N-16P_XP_001742640.1_PDZ_1	MB2 *M.brevicollis* PDZ 1
MB2_MBrevicollis_4P-S-N-16P_XP_001742640.1_PDZ_0	MB2 *M.brevicollis* PDZ 0
MB2_MBrevicollis_4P-S-N-16P_XP_001742640.1_PDZ_-1	MB2 *M.brevicollis* PDZ -1
SR4_SRosetta_SRosetta_3P-S-N-13P_XP_004996158.1_PDZ_3	SR4 *S.rosetta* PDZ 3
SR4_SRosetta_SRosetta_3P-S-N-13P_XP_004996158.1_PDZ_2	SR4 *S.rosetta* PDZ 2
SR4_SRosetta_SRosetta_3P-S-N-13P_XP_004996158.1_PDZ_1	SR4 *S.rosetta* PDZ 1

Supplementary Table 4: Naming conventions and accession numbers of sh3+NK genes and their corresponding PDZ domains in Supplementary Figure 3.

**Simulation (Protein x Ligand 10 aa)**	**ΔG_bind Free Energy (REU)**	**ΔG_bind Potential Energy (kJ/mol)**
AQ PDZ3 x AQ CRIPT Average	-60.08 ± 0.38	-845.94 ± 38.19
CO1 PDZ3 x CO CRIPT Average	-58.26 ± 0.79	-753.71 ± 89.61
CO2 PDZ x CO CRIPT Average	-74.07 ± 0.60	-709.87 ± 126.32
HS PDZ3 x HS CRIPT average	-81.32 ± 1.95	-1258.72 ± 96.81
PDZ3 x VANGL2	-58.55 ± 2.31	-845.52 ± 126.32
PDZ3 x KALIRIN7	-57.39 ± 6.96	-477.35 ± 97.36
PDZ3 x CITRON	-61.72 ± 1.55	-860.87 ± 90.00
PDZ3 x SYNGAP	-66.23 ± 1.19	-1077.61 ± 44.65
PDZ3 x HEXAPEPTIDE	-60.27 ± 3.25	-842.97 ± 52.04
HS PDZ 3 x HS NMDA	-49.29 ± 0.76	-747.58 ± 101.58
PDZ3 x GGGGGGGGGG	-33.82 ± 0.81	-546.60 ± 57.24
PDZ3 x AAAAAAAAAA	-48.64 ± 2.72	-730.04 ± 70.23
PDZ3 x TNF	-65.01 ± 1.30	-726.63 ± 66.78
PDZ3 x endozepine	-67.04 ± 1.83	-891.62 ± 60.24
PDZ3 x C-C motif chemokine 4 precursor	-59.17 ± 1.09	-384.80 ± 162.16

Supplementary Table 5: The intermolecular potential energy between each PDZ protein and its ligand.
